# Ethnic differences in dissatisfaction with sexual life in patients with type 2 diabetes in a Swedish town

**DOI:** 10.1186/1471-2458-10-536

**Published:** 2010-09-08

**Authors:** Marina Taloyan, Alexandre Wajngot, Sven-Erik Johansson, Jonas Tovi, Jan Sundquist

**Affiliations:** 1 Center for Primary Health Care Research, Region Skåne, Lund University, Lund, Sweden; 2 Karolinska Institutet, Center for Family and Community Medicine, Stockholm, Sweden; 3 Stanford Prevention Research Center, Stanford University School of Medicine, Stanford, USA

## Abstract

**Background:**

The first aim of this study was to analyze whether self-reported satisfaction with one's sexual life was associated with ethnicity (Swedish and Assyrian/Syrian) in patients with type 2 diabetes. The second was to study whether the association between satisfaction with one's sexual life and ethnicity remained after controlling for possible confounders such as marital status, HbA1c, medication, and presence of other diseases.

**Methods:**

This cross-sectional, questionnaire-based study was conducted at four primary health care centers in the Swedish town of Södertälje. A total of 354 persons (173 ethnic Assyrians/Syrians and 181 ethnic Swedes) participated.

**Results:**

The total prevalence of self-reported dissatisfaction with one's sexual life in both groups was 49%. No significant ethnic differences were found in the outcome. In the final model, regardless of ethnicity, the odds ratio (OR) for self-reported dissatisfaction with one's sexual life in those ≥ 70 years old was 2.52 (95% CI 1.33-4.80). Among those living alone or with children, the OR was more than three times higher than for married or cohabiting individuals (OR = 3.10, 95% CI 1.60-6.00). Those with other diseases had an OR 1.89 times (95% CI 1.10-3.40) higher than those without other diseases.

**Conclusions:**

The findings demonstrate that almost half of participants were dissatisfied with their sexual life and highlight the importance of sexual life to people with type 2 diabetes. This factor should not be ignored in clinical evaluations. Moreover, the findings demonstrate that it is possible to include questions on sexual life in investigations of patients with type 2 diabetes and even in other health-related, questionnaire studies, despite the sensitivity of the issue of sexuality.

## Background

Sexual problems are common complications of diabetic disease in both men and women [1-6]. In general, the prevalence of sexual dysfunction increases with age [7-9] and in the presence of cardiovascular disease [10,11]. Furthermore, changes in hormones and endocrine function [12], biochemical factors (HbA1c, triglycerides), and psychosocial factors [13-16] can also have an impact on sexual function.

Few studies have investigated sexuality in people from different ethnic groups who have diabetes. An explorative Swedish study by Hjelm et al. concluded that there were different beliefs about health and illness in men with diabetes in different ethnic groups. According to the results of this study, Swedes focused on heredity, lifestyle, and management of diabetes, while sexual function was reported as one of the factors important to health among Arabs and men from the former Yugoslavia [17]. Few studies address female sexuality and sexual expression in immigrant women with diabetes. For cultural reasons and because of methodological difficulties, the association between type 2 diabetes and sexual function in women has been studied in fewer objective studies than the effects of diabetes on sexual function in men [4].

Although we believed that questions regarding sexual life would be sensitive, we included three questions about this matter in the questionnaire used to investigate the health and well-being of patients with type 2 diabetes in the town of Södertälje. The aim was to analyze whether self-reported satisfaction with one's sexual life was associated with ethnicity (Swedish and Assyrian/Syrian) in patients with type 2 diabetes and when taking age and biological sex into consideration. We also wanted to study if any such association between satisfaction with one's sexual life and ethnicity remained when possible confounders such as marital status, HbA1c, medication, and other diseases were taken into consideration.

## Methods

### Sample

The participants were selected from the registers of patients with type 2 diabetes at four primary health care centers in the town of Södertälje. A total of 354 individuals were included: ethnic Assyrians/Syrians (n = 173) and ethnic Swedes (n = 181). They were matched according to gender and age. Those who identified themselves as neither Assyrian/Syrian nor Swedish were excluded from the study. Ethnicity is discussed further in the section on explanatory variables below.

The patients were contacted by telephone and later filled out a questionnaire at their health care center in the presence of an interviewer (the first author or a GP), who helped with the questions as needed. Interpreters were also used as needed. Medical information and laboratory data from the patient records of all participants were gathered. Verbal informed consent was obtained for participation in the study and for the gathering of data from patient records.

The 61 participants who did not answer the question about the level of satisfaction with their sexual life and the two participants who identified themselves as neither ethnic Assyrians/Syrians nor ethnic Swedes were excluded from the study, leaving a total of 291 participants: 129 Assyrians/Syrians and 162 Swedes.

### Outcome variable

Information on the outcome variable, *self-reported dissatisfaction with one's sexual life*, was obtained from the answers to one of three questions on sexual life asked as part of the larger structured health questionnaire developed specifically for this study. The outcome of the study is based on the question: "Are you satisfied with your sexual life?" Another two questions (concerning loss of ability to have sexual intercource and loss of sexual desire) are not included in this study. Five answers were possible: "very dissatisfied," "quite dissatisfied," "rather satisfied," "quite satisfied", and "very satisfied". Those who reported that they were "very dissatisfied," "quite dissatisfied," or "rather satisfied" were categorized as having *self-reported dissatisfaction with their sexual life*.

Before asking the participants the three questions about satisfaction/dissatisfaction with their sexual life, we informed them: "The following questions can be perceived as sensitive; please answer as well as you can." This information was provided on the questionnaire and verbally, as necessary, for clarification. Verbally, the interviewers also explained that the reason we were asking these questions was that type 2 diabetes can have complications that may affect one's sexual life.

### Explanatory variables

The variable *Self-reported ethnicity *included two groups: Assyrians/Syrians and Swedes. Assyrians/Syrians are an ancient ethnic group from Mesopotamia whose Christian religion is an important part of their identity [18]. There are approximately 70,000 to 80,000 Assyrians/Syrians in Sweden [18], originating mainly from Turkey, Syria, Iraq, and Lebanon. About 20,000 Assyrians/Syrians live in Södertälje.

Assyrians belong to one of four churches: the Syrian-Orthodox, the Nestorian, the Chaldeian, and the Syrian-Catholic. Individuals from the Syrian-Orthodox group and those who do not want to be defined as Assyrian may identify themselves as Syrian [19]. Thus, both terms were used in the questionnaire.

Official Swedish statistics do not include information on ethnicity; rather, persons are identified by country of birth, parents' country of birth, and citizenship. For this reason, the identification of potential participants by ethnicity took place in two stages. To begin with, the first author and personnel from the primary health care centers in Södertälje examined the list of patients with diabetes from each of the four participating primary health care centers. Using participants' surnames and the health care center personnel's personal knowledge of patients, the first author and the health care center personnel identified persons they believed to be ethnic Assyrians/Syrians or Swedes. The first author then developed a sex- and age-matched list of prospective Assyrian/Syrian and Swedish responders. Health care center personnel contacted prospective responders by phone and invited them to fill out the questionnaire at the primary health care center.

One of the questions on the questionnaire was about ethnicity. The Assyrian/Syrian participants all came from Middle Eastern countries: 33.5% from Turkey, 30.6% from Iraq, 20.2% from Syria, and the remainder from Lebanon and other countries.

*Age *was divided into three groups: 32-59, 60-69 and ≥ 70 years, to obtain similar proportions of patients in each group.

*Marital status *was divided into two groups. The first consisted of those who were married or cohabiting, and the second consisted of both those who lived without a wife/husband, whether living alone or with their children, because of the small sample size of participants in the latter group.

*HbA1c *was divided into two groups: normal (≤ 6.0%) and high (< 6.0%). HbA1c was measured using the Swedish mono-S method [20].

*Medication *was based on the question "Do you take blood sugar reducing medicine and/or insulin? There were two response alternatives "yes. Which?" and "no". Positive responses were considered as having medication (drugs and/or insulin) and all negative answers were classified as not having medication.

*Other disease(s) *was defined on the basis of the question, "Do you have other diseases? (Yes/No)". All "yes" answers were categorized as having other disease(s) and all "no" answers were categorized as not having other disease(s).

### Statistical analyses

Pearson's chi-2 test and the t-test were used to calculate whether differences between the two ethnic groups in the prevalence of sociodemographic characteristics were statistically significant. Prevalence of the outcome variable was calculated in the same way. Unconditional logistic regression was used to calculate the odds ratios (ORs) and 95% confidence intervals (95% CIs) to investigate the relation between self-reported dissatisfaction with one's sexual life and the explanatory variables. All explanatory variables, which included ethnicity, sex, age (years), marital status, HbA1c, medication, and other diseases were introduced stepwise into the models. The fit of the models was judged by the Hosmer-Lemeshow goodness-of-fit test. The models were considered acceptable at p > 0.05, and both models met this demand [21]. The statistical software program used was Stata v.9 [22].

### Ethical considerations

The study was approved by the Regional Ethical Committee of Karolinska Institutet (reference number 2006/4:8, 2006-09-27).

## Results

The questionnaire/interview was completed by 354 participants. Of these participants, 83% (n = 291) responded to the question about satisfaction with their sexual life. The response rate was 75% among the Assyrians/Syrians and 90% among the Swedes. In total, 94% of men (62.8% of Assyrian/Syrian men and 60.5% of Swedish men) and 68% of women (37.2% of Assyrian/Syrian women and 39.5% of Swedish women) answered the question. A comparison of nonresponders in both ethnic groups showed no significant differences with regard to such explanatory variables as marital status.

There were statistically significant differences between the ethnic groups with regard to a number of explanatory variables (Table 1). In comparison to the Swedes, the Assyrians/Syrians were younger, fewer lived alone or with children (8.5% vs. 37.0%), and a significantly higher number had abnormal HbA1c.

**Table 1:** Distribution (%) of the explanatory variables by ethnicity (n = 291)

Variable	Ethnicity		
	Assyrian/Syrian (n = 129)	Swedish (n = 162)	Test of difference
			
Gender			
Female	37.2	39.5	
Male	62.8	60.5	
			
Age (years)			0.000
32-59	57.4	33.3	
60-69	22.5	34.0	
≥ 70	20.1	32.7	
			
Marital status			0.000
Married/cohabiting	91.5	63.0	
Living alone/with children	8.5	37.0	
			
Abnormal HbA1c	50.0	37.2	0.030
			
Medication (yes)	78.0	79.0	0.890
			
Other diseases	70.8	71.4	0.918

The total prevalence of self-reported dissatisfaction with one's sexual life in both groups was 49% (not shown in table). No significant difference in this self-reported dissatisfaction was noted between the ethnic groups; as shown in Table 2, a total of 46.5% of the Assyrians/Syrians and 51.0% of the Swedes reported dissatisfaction. In both ethnic groups, more participants who lived alone or with children were dissatisfied than those who were married or cohabiting, but in the Assyrian/Syrian group, only 8.5% of the participants lived alone or with children. The responders in the oldest group (≥ 70 years) were the next most dissatisfied group.

**Table 2:** Prevalence (%) of self-reported dissatisfaction with one's sexual life by the explanatory variables and ethnicity (n = 291)

Variable	Ethnicity		
	**Assyrian/Syrian** **(n = 129)**	**Swedish** **(n = 162)**	**Test of difference**

Total	46.5	51.0	NS
			
Gender			NS
Female	54.2	45.3	
Male	42.0	54.1	
			
Age (years)			NS
32-59	40.5	42.6	
60-69	51.7	50.0	
≥ 70	57.7	60.4	
			
Marital status			NS
Married/cohabiting	43.2	43.0	
Living alone/with children	81.8	61.0	
			
Abnormal HbA1c			NS
Yes	51.6	48.3	
No	42.2	52.0	
			
Medication			NS
Yes	46.1	49.0	
No	48.0	55.2	
			
Other diseases			NS
Yes	49.0	57.1	
No	40.5	35.7	

Table 3 shows the association between ethnicity and self-reported dissatisfaction with one's sexual life in two models: Model 1, adjusted for age and biological sex, and Model 2, adjusted for age, biological sex, marital status, HbA1c, medication, and presence of other diseases. There were no statistically significant differences between Assyrians/Syrians and Swedes with regard to the outcome variable in Model 1 or 2. No significant differences with regard to the outcome variable were found by biological sex in either model. The ORs for dissatisfaction with one's sexual life in those ≥ 70 years old was significantly higher (OR = 2.40, 95%, CI 1.24-4.52) than for those in the younger age groups (OR = 1.54, 95% CI 0.83-2.84) in Model 1. In Model 2, the OR increased in those ≥ 70 years old (OR= 2.80, 95% CI 1.40-5.54) and remained statistically significant. The OR for self-reported dissatisfaction with one's sexual life among those living alone or with children was approximately three times higher than for married or cohabiting individuals (OR = 2.94, 95% CI 1.44-6.01). The OR for reporting sexual dissatisfaction was twice (95% CI 1.10-3.60) higher among those with other diseases than those without other diseases.

**Table 3:** The odds ratios (ORs) with 95% confidence intervals (95% CIs) for self-reported dissatisfaction with one's sexual life

Variable	Model 1*	Model 2**
Ethnicity		
		
Swedish-born	1	1
Assyrian/Syrian	0.94 (0.55-1.60)	0.70 (0.40-1.25)
		
Gender		
Male	1	1
Female	1.15 (0.70-1.94)	0.96 (0.54-1.70)
		
Age (years)		
32-59	1	1
60-69	1.54 (0.83-2.85)	1.52 (0.80-2.96)
≥ 70	**2.40** **(1.24-4.52)**	**2.80** **(1.40-5.54)**
		
Marital status		
Married/cohabiting		1
Living alone/with children		**2.94** **(1.44-6.01)**
		
HbA1c		
Normal		1
Abnormal		1.28 (0.72-2.30)
		
Medication		
No		1
Yes		0.70 (0.33-1.35)
		
Other diseases		
No		1
Yes		**2.00** **(1.10-3.60)**

*Model 1 is adjusted for age and biological sex.

**Model 2 is adjusted for all explanatory variables.

## Discussion

The main finding of this study is that the total prevalence of reported dissatisfaction with one's sexual life in the study population is about 50%. Furthermore, there were no significant differences by ethnicity in the prevalence or ORs of reporting dissatisfaction. Instead, the factors older age, living alone or with children, and presence of other disease(s) were significantly and independently associated with the outcome variable.

Age in and of itself is a risk factor for both poorer general health [23,24] and type 2 diabetes [2]. Our findings regarding people aged ≥ 70 years are consistent with the findings of other studies on negative effects of diabetes on the sexuality of elderly persons [2,24].

Although it might seem to be a self-evident topic for research, the association between marital status and satisfaction with sexual life in persons with diabetes has not been well-researched. A study exploring the relationship between marital relational domains such as intimacy and adjustment in insulin-treated adults with type 1 and type 2 diabetes concluded that the quality of marriage was associated with adaptation to diabetes [25]. Another study investigated sexual and marital satisfaction in 26 men with diabetes and 30 men with spinal cord injury who received penile prostheses or injections for the treatment of impotence. Both groups reported more disagreements with their partners about factors of importance in their relationship than a normative sample but engaged in more and more frequent sexual activity with their partners after treatment regardless of which kind of treatment they received [26].

These studies focused on satisfaction with sexual life in married persons with diabetes. Our findings indicate that persons who were living alone or with children had three time's higher odds of reporting dissatisfaction with their sexual life than those who were married or cohabiting. Further research on satisfaction with one's sexual life could therefore focus on persons with type 2 diabetes who are living alone.

Previous studies have established that individuals with diabetes who rate their health as poor often have other diseases in addition to diabetes [23,24,27]. The question of whether the primary risk factor is type 2 diabetes, complications of type 2 diabetes, or other coexisting health factors constitutes the ground for ongoing and future investigation and discussion.

In general, individuals' sexual life can be influenced by several health and sociodemographic factors. Previous studies indicate that sexual dissatisfaction, lack of desire, low sexual arousal/erection, and pain during intercourse can negatively affect the sexual life of persons with type 2 diabetes [28,29]. A study of 230 married Malaysian women from the general population showed that female sexual dysfunction was associated with such sociodemographic and health factors as being married more than 14 years, older age, being Malay, having less frequent sexual intercourse, having more children, having a higher academic status, and lack of lubrication [30].

Dissatisfaction with one's sexual life can be associated with physiological and psychological factors. For instance, the association between poor life satisfaction and poor general health is known to be strong. In one Swedish study [31], subjects with poor life satisfaction had a higher risk of reporting poor health (OR = 15) than those who reported higher life satisfaction. The authors of the same study reported that country of birth, depression, and reporting many symptoms were related to poor health. Another study based on a nationally representative Swedish sample investigating life satisfaction found that having no partner and being a first-generation immigrant were associated with reporting of low levels of satisfaction [32]. We had no data on life satisfaction in the present study, but it is possible that satisfaction with one's sexual life has a strong influence on both life satisfaction and self-reported general health status.

The culture of the responders may have influenced their answers to questions about sexual life, which can be perceived as being sensitive. It is important to note that individuals with an Assyrian/Syrian ethnic background reported sexual life mostly within marriage. This is probably because of cultural and religious values; for example, in women [19,33]. The majority of Assyrian/Syrian participants in the present study stated that they accepted type 2 diabetes as something sent by God, and separation or the loss of one's partner was not followed by the choice of another sex partner. "We do not do that," said a separated 40-year-old woman when we asked if she had a sexual partner. We believe that these culturally influenced beliefs and choices may be one of the reasons why a smaller percentage of Assyrian/Syrian participants (75%) than Swedish ones (90%) responded to this question. Sixty-nine percent of all the nonresponding women had an Assyrian/Syrian ethnic background. It is also possible that for cultural or religious reasons, sexual life may not be an important part of some individuals' lives, which means that not having an active sexual life might be satisfactory to them.

Health-related questionnaires sometimes omit relevant questions about sexual life, often because such questions are considered private, sensitive, or even taboo for cultural or religious reasons. However, we believe that it is possible to ask such questions if they are carefully worded. Furthermore, we believe that it is important to ask such questions because sexuality is an important part of health and the quality of life. In this paper, we chose to focus on one of the three questions about sexual life in our questionnaire: "Are you satisfied with your sexual life?" (see additional file 1). This question was chosen because we regarded the inclusion of this general question as a first step in including questions on sexual life in studies on the care of persons with diabetes.

This study has several strengths. To the best of our knowledge, this is one of very few questionnaire-based health studies in Sweden to gather information about people's self-reported satisfaction with their sexual life, a factor that impacts both health and the quality of life.

Moreover, there is noregistration by ethnicity in the official Swedish statistics, in which persons are identified by country of birth, parents' country of birth, and citizenship. Thus, a unique aspect of this study is the use of data on self-reported ethnicity. The main strength of the study is that it is based on the first cross-sectional survey of a representative sample of ethnic Assyrian/Syrian immigrants to Sweden with type 2 diabetes. Although the sample in the study is small, approximately 20,000 of the total 70,000 to 80,000 Assyrians/Syrians in Sweden live in the town of Södertälje.

A major limitation of this study is that the sample represents Assyrian/Syrian patients with type 2 diabetes living in one town, so the results cannot be generalized to the entire population of Assyrians/Syrians with diabetes in Sweden. Another limitation is that the cross-sectional nature of the study and the small sample size preclude the possibility of drawing extensive causal conclusions. An additional limitation of this study is the use of a questionnaire that was not validated. Another limitation is that the range of ages in the youngest of the three age groups (32-59 years) was much broader that the range of ages in the middle group (60-69 years). We chose to divide age in this way to obtain similar proportions of patients in each age group, which is adequate for statistical analyses. A further limitation is that we invited particular groups to participate in the study, which may have resulted in selection bias. Furthermore, in this study, we did not ask about menopause. We also lacked detailed information on "other diseases" because data on this issue was gathered via only one question, "Do you have any other diseases?" The response alternatives were "yes, which one or ones?" or "no" and we lack the answers about which other diseases the participants had. Moreover, we did not take the information on diabetes complications into account. Finally, the small number in the oldest age range in the Assyrian/Syrian group is also a limitation.

## Conclusion

No differences in self-reported dissatisfaction with one's sexual life were observed between Assyrian/Syrian and Swedish patients with type 2 diabetes. Nevertheless, the findings of this study suggest that other factors may play an important role in satisfaction with one's sexual life, including age, marital status, and the presence of other diseases in addition to diabetes.

The high prevalence of dissatisfaction with sexual life reported by responders highlights the importance of sexual life to people with type 2 diabetes, so that this factor should not be ignored in clinical evaluations. Moreover, the findings demonstrate that it is possible to include questions on sexual life in investigations of patients with type 2 diabetes as well as in other health-related, questionnaire studies, despite the sensitivity of the issue of sexuality. We found that the key to asking such questions successfully was to take into account individuals' cultural and religious identities in order to find the best way to ask questions about sensitive or even such taboo topics as sexual life.

## Competing interests

The authors declare that they have no competing interests.

## Authors' contributions

MT planned and conducted the study and drafted the first version of the manuscript. MT and SEJ performed the statistical analysis. JT and AW contributed to the discussion section. JS read and approved the final manuscript. All authors read and approved the final manuscript.

## Pre-publication history

The pre-publication history for this paper can be accessed here:

http://www.biomedcentral.com/1471-2458/10/536/prepub

## Supplementary Material

Additional file 1 **Questions included in the present study**. The file contains 8 questions that were asked as part of the original 52-item questionnaire. The answers to the 8 questions were used as the basis for the outcome and explanatory variables. Click here for file
