# Supplementary material for: Ethnic differences in dissatisfaction with sexual life in patients with type 2 diabetes in a Swedish town
Source: BMC Public Health. 2010 Sep 8;10:536. doi: 10.1186/1471-2458-10-536 (PMC2944369; doi:10.1186/1471-2458-10-536)
Supplement: Additional file 1 — Questions included in the present study. The file contains 8 questions that were asked as part of the original 52-item questionnaire. The answers to the 8 questions were used as the basis for the outcome and explanatory variables. [file 1471-2458-10-536-S1.DOC]

**Questions included in the present study:**

When were you born? Year___

Who are included in your household?

- 1. I live alone
  2. I live with: Husband/Wife; Children; Sibling/s; Other/s_________

Were you a member of some ethnic, linguistic, or religious minority in your country of birth?

Yes. Which one? _____ No.

How many years of formal education do you have?_____

Do you know your medical test results, such as:

HbA1c, how high? ___

Blood pressure, how high?___________

Cholesterol and triglycerides? _______

Do you have other diseases? Yes. Which one or ones? ____No

Do you take oral blood sugar reducing medicine and/or insulin?

Yes. Which one or ones?____ No

How satisfied are you with your sexual life? (Choose one alternative)

Very dissatisfied

Rather dissatisfied

Neither satisfied nor dissatisfied

Rather satisfied

Very satisfied
